# *Kaempferia parviflora* Rhizome Extract Improves Skeletal Muscle Glucose Homeostasis by Upregulating the GLUT4 Signaling Pathway In Vitro and In Vivo

**DOI:** 10.3390/ph19050754

**Published:** 2026-05-12

**Authors:** Seong-Hoo Park, Yeonhwa Lee, Soo-Jeung Park, Hyunyoung Choi, Jeongjin Park, Jinhak Kim, Kwang-Soo Baek, Kun Hee Park, Woojin Jun

**Affiliations:** 1 Research Institute of Medical Nutrition, Kyung Hee University, Seoul 02447, Republic of Korea; phoo3166@khu.ac.kr (S.-H.P.); hyun00807@khu.ac.kr (H.C.); 2 Division of Food and Nutrition, Chonnam National University, Gwangju 61186, Republic of Korea; qazwsx917@naver.com (Y.L.); pjj8425@hanmail.net (J.P.); 3 Department of Culinary and Nutrition, Woosong University, Daejeon 34606, Republic of Korea; sjpark07@wsu.ac.kr; 4 Research Institute for Human Ecology, Chonnam National University, Gwangju 61186, Republic of Korea; 5 R&D Division, Daehan Chemtech Co., Ltd., Gwacheon 13840, Republic of Korea; jhkim@dhchemtech.com (J.K.); rnd@dhchemtech.com (K.-S.B.); soske0328@gmail.com (K.H.P.)

**Keywords:** *Kaempferia parviflora*, C2C12, db/db mice, diabetes, GLUT4, glucose homeostasis

## Abstract

**Background/Objectives:** *Kaempferia parviflora* (KP), also known as black ginger, has been reported to exert metabolic regulatory effects, yet its influence on skeletal muscle glucose handling remains unclear. Because impaired glucose uptake in skeletal muscle is a major contributor to insulin resistance and type 2 diabetes mellitus (T2DM), this study investigated the effects of KP on glucose uptake and the GLUT4 signaling pathway both in vitro and in vivo. **Methods:** The effects of KP extract on glucose uptake and GLUT4-related signaling proteins were evaluated in differentiated C2C12 myotubes. In parallel, C57BL/6J and db/db mice were orally administered KP extract at varying doses for six weeks to evaluate its metabolic effects in vivo. **Results:** KP significantly increased glucose uptake in C2C12 myotubes, accompanied by increased GLUT4 expression and upregulation of GLUT4-related signaling proteins. In diabetic mice, KP administration improved fasting glucose, glucose tolerance and GLP-1 level while lowering insulin and HbA1c levels. Skeletal muscle tissues exhibited increased GLUT4 expression and modulation of downstream regulators involved in glucose homeostasis. **Conclusions:** These findings indicate that KP improves skeletal muscle glucose metabolism primarily through activation of the GLUT4 pathway. Taken together, KP may serve as a potential functional ingredient for regulating muscle glucose utilization and mitigating metabolic disturbances associated with T2DM.

## 1. Introduction

Diabetes mellitus (DM) is a major global health problem that continues to increase in prevalence and contributes to substantial morbidity and mortality through complications such as nephropathy, neuropathy, cardiovascular dysfunction, and retinopathy [1,2]. Among its forms, type 2 diabetes mellitus (T2DM) accounts for the vast majority of cases and is primarily characterized by insulin resistance, impaired glucose uptake, and dysregulated energy metabolism [3]. Although numerous pharmacological agents, including metformin, thiazolidinediones, and GLP-1 analogs, are widely used, many patients fail to achieve sustained glycemic control and often experience undesirable side effects [4,5]. This has heightened interest in natural product-derived compounds that can safely modulate glucose metabolism and improve insulin sensitivity [6].

Skeletal muscle is the principal site of postprandial glucose disposal, accounting for nearly 80% of insulin-stimulated glucose uptake [7]. In healthy individuals, insulin triggers a cascade involving the insulin receptor, insulin receptor substrate-1 (IRS-1), phosphoinositide 3-kinase (PI3K), and protein kinase B (Akt), ultimately promoting the translocation of the glucose transporter GLUT4 to the plasma membrane [8]. In T2DM, defects in this signaling pathway lead to reduced GLUT4 expression, diminished glucose uptake, and progressive insulin resistance [9]. Because skeletal muscle plays such a pivotal role in systemic glucose homeostasis, therapeutic strategies targeting GLUT4-mediated glucose transport mechanisms in muscle are considered essential for improving metabolic control in diabetic conditions [10]. The db/db mouse, deficient in the leptin receptor, represents one of the most widely used T2DM models and exhibits hyperphagia, obesity, hyperglycemia, and impaired muscle insulin signaling [11]. These pathological characteristics closely mimic human metabolic dysfunction, making the model highly suitable for evaluating muscle-specific glucose regulatory mechanisms [10,11].

*Kaempferia parviflora* (KP), commonly known as black ginger, has long been consumed in Southeast Asian traditional medicine for enhancing vitality, circulation, and endurance [12,13]. Recent studies suggest that its unique methoxyflavones possess diverse biological properties, including anti-inflammatory, antioxidant, and energy-modulating effects [14,15]. In particular, KP has been reported to promote energy metabolism and exercise performance through activation of brown adipose tissue and upregulation of UCP-1 [16], while improving skeletal muscle function and endurance via the SIRT1/AMPK/PGC-1α signaling pathway [17,18,19]. In addition, we previously showed that the same standardized KPE exerts significant anti-obesity effects in high-fat diet (HFD)-fed mice by suppressing adipogenesis and lipogenesis while stimulating lipolysis and thermogenesis in adipose tissue [19]. Despite these well-established metabolic benefits, the specific role of KP in regulating glucose handling in skeletal muscle remains poorly understood particularly regarding its influence on the GLUT4 signaling axis, a central pathway governing insulin stimulated glucose uptake and metabolic homeostasis. Elucidating whether KP modulates GLUT4-related signaling may provide mechanistic insight into its potential as a nutritional intervention for improving skeletal muscle glucose metabolism. Importantly, emerging evidence indicates that KP may exert beneficial actions on skeletal muscle function, mitochondrial biogenesis, and physical performance [17,18]. However, its specific effects on glucose handling in muscle tissue—particularly its influence on the GLUT4 signaling axis—remain insufficiently understood.

In this study, we investigated the effects of KP on glucose uptake and GLUT4-related signaling using C2C12 myotubes as an in vitro model of skeletal muscle glucose metabolism. Additionally, we evaluated whether chronic oral administration of KP improves metabolic parameters and muscle glucose regulation in db/db diabetic mice. By elucidating its molecular actions on the GLUT4 pathway, this study aims to provide new insight into the potential of KP as a functional natural compound for improving skeletal muscle glucose utilization and mitigating insulin resistance in T2DM.

## 2. Results

### 2.1. Kaempferia parviflora Extract (KPE) Inhibits α-Amylase and α-Glucosidase Activities In Vitro

**Figure 1:**
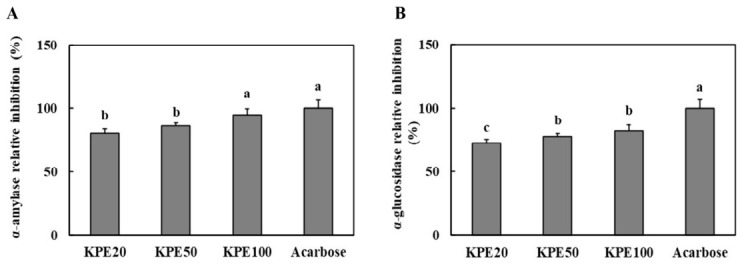
*Kaempferia parviflora* extract (KPE) significantly inhibits α-amylase and α-glucosidase activities. (**A**) α-Amylase inhibitory activity; (**B**) α-glucosidase inhibitory activity. KPE 20, KPE at 20 μg/mL; KPE 50, KPE at 50 μg/mL; KPE 100, KPE at 100 μg/mL; Acarbose, acarbose at 100 μg/mL. Values are presented as mean ± standard deviation (n = 3). Different superscript letters indicate significant differences at *p* < 0.05.

To evaluate the inhibitory effect of KPE on carbohydrate-digesting enzymes, α-amylase and α-glucosidase inhibition assays were performed. As shown in Figure 1A, KPE treatment significantly suppressed α-amylase activity in a dose-dependent manner. KPE at 100 μg/mL exhibited the highest inhibitory effect among the tested concentrations. Statistically, the inhibitory activity of KPE 100 μg/mL was comparable to that of the positive control, acarbose, with no significant difference observed between the two.

Similarly, KPE markedly inhibited α-glucosidase activity (Figure 1B). While the inhibitory effect of KPE 100 μg/mL was potent, it was significantly lower than that of acarbose (*p* < 0.05), which demonstrated the strongest inhibition overall. The inhibitory effect increased with concentration, with KPE 100 μg/mL showing significantly greater inhibition than KPE 20 μg/mL and KPE 50 μg/mL (*p* < 0.05). Acarbose demonstrated the strongest inhibition overall, as expected; however, KPE 100 μg/mL produced a comparable level of suppression.

These results indicate that KPE possesses substantial inhibitory activity against both α-amylase and α-glucosidase, suggesting its potential to reduce postprandial glucose elevation by modulating carbohydrate digestion.

### 2.2. KPE Attenuates Palmitic Acid-Induced Impairment of Glucose Uptake in C2C12 Myotubes

**Figure 2:**
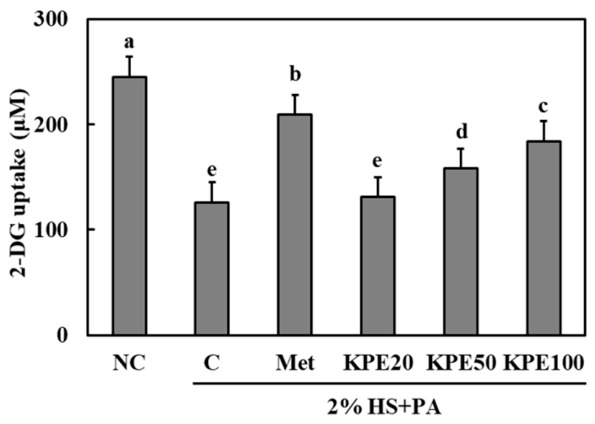
*Kaempferia Parviflora* extract (KPE) increases glucose uptake in C2C12 myotubes under insulin-present conditions. C2C12 myotubes were co-treated with palmitic acid (PA) and KPE during differentiation (day 3–5), followed by insulin stimulation (1 μM, 20 min), which was applied uniformly to all groups, prior to glucose uptake measurement. NC, normal control; C, differentiation-induced control treated with 2% horse serum (HS) and PA; Met, PA-induced cells treated with metformin (1 mM); KPE 20, PA-induced cells treated with KPE at 20 μg/mL; KPE 50, PA-induced cells treated with KPE at 50 μg/mL; KPE 100, PA-induced cells treated with KPE at 100 μg/mL. Values are presented as mean ± standard deviation (n = 3). Different superscript letters indicate significant differences at *p* < 0.05.

To investigate whether KPE modulates glucose uptake under lipotoxic conditions, we measured 2-DG uptake in differentiated C2C12 myotubes treated with palmitic acid (PA). As shown in Figure 2, the normal control (NC) group exhibited the highest level of glucose uptake, whereas PA-treated control (C) group showed a significant reduction in uptake (*p* < 0.05). Metformin treatment (Met) group partially restored glucose uptake, resulting in levels considerably higher than those of the PA-treated groups.

KPE administration also improved glucose uptake in a concentration-dependent manner. While KPE 20 μg/mL did not significantly differ from the C group, treatment with 50 μg/mL and 100 μg/mL markedly increased glucose uptake relative to the C group (*p* < 0.05). Notably, KPE 100 μg/mL demonstrated a substantial improvement, approaching the response observed in the metformin-treated cells.

These findings suggest that KPE mitigates PA-induced insulin resistance in skeletal muscle cells by enhancing glucose uptake, highlighting its potential role in improving muscle glucose metabolism.

### 2.3. KPE Ameliorates PA-Induced Impairment of Insulin Signaling and GLUT4 Expression in C2C12 Myotubes

**Figure 3:**
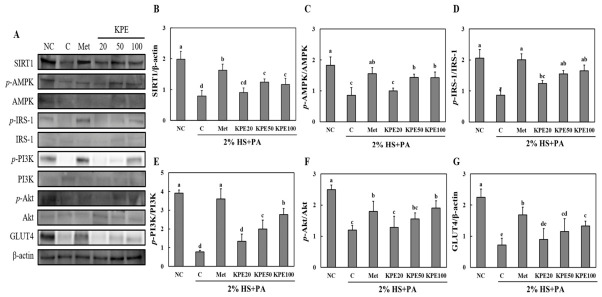
*Kaempferia parviflora* extract (KPE) upregulates GLUT4 expression in differentiated C2C12 myotubes. (**A**) Representative immunoblots; (**B**) SIRT1; (**C**) *p*-AMPK/AMPK; (**D**) *p*-IRS-1/IRS-1; (**E**) *p*-PI3K/PI3K; (**F**) *p*-Akt/Akt; (**G**) GLUT4 protein expression. NC, normal control; C, differentiation-induced control treated with 2% horse serum (HS) and palmitic acid (PA); Met, PA-induced cells treated with metformin (1 mM); KPE 20, PA-induced cells treated with KPE at 20 μg/mL; KPE 50, PA-induced cells treated with KPE at 50 μg/mL; KPE 100, PA-induced cells treated with KPE at 100 μg/mL. Values are presented as mean ± standard deviation (n = 3). Different superscript letters indicate significant differences at *p* < 0.05.

To further elucidate the molecular mechanisms underlying the improvement of glucose uptake by KPE, we examined key proteins involved in insulin signaling pathways in PA-treated C2C12 myotubes. As shown in Figure 3A–G, PA exposure markedly reduced the expression of sirtuin 1 (SIRT1), phosphorylated AMP-activated protein kinase (AMPK), phosphorylated IRS-1, phosphorylated PI3K, phosphorylated Akt, and GLUT4 compared with the normal control group (*p* < 0.05).

Metformin treatment significantly restored the expression of SIRT1, phosphorylation of AMPK, IRS-1, PI3K, and Akt, as well as GLUT4 expression, confirming its expected insulin-sensitizing effects. KPE treatment also improved these signaling proteins in a dose-dependent manner. KPE 50 μg/mL and 100 μg/mL significantly increased SIRT1 expression and AMPK phosphorylation compared to the PA-treated control (*p* < 0.05). Moreover, phosphorylation levels of IRS-1, PI3K, and Akt were notably elevated in the KPE 50 μg/mL and 100 μg/mL groups, indicating partial restoration of insulin signaling under lipotoxic conditions.

Consistent with these upstream improvements, GLUT4 expression was significantly increased following KPE treatment, with the highest expression observed in the KPE 100 μg/mL group. These results suggest that KPE enhances insulin signaling and promotes GLUT4-mediated glucose transport mechanism by activating the SIRT1/AMPK and PI3K/Akt pathways in PA-induced insulin-resistant muscle cells.

### 2.4. Effects of KPE Supplementation on Body and Organ Weights in High-Fat Diet (HFD)-Fed db/db Mice

KPE supplementation exerted notable effects on body weight regulation in HFD-fed db/db mice (Table 1). As expected, the diabetes-induced control (C) group exhibited significantly higher final body weight and weight gain over 6 weeks compared with the normal control (NC) group (*p* < 0.05). Metformin-treated mice (Met) showed a moderate reduction in weight gain relative to the diabetes control group, confirming the responsiveness of this model.

KPE administration led to a dose-dependent improvement in weight parameters. Mice receiving 40 mg/kg KPE exhibited significantly lower final body weight and weight gain compared with the diabetes control group (*p* < 0.05), whereas the 20 mg/kg KPE group showed a decreasing trend without reaching statistical significance. The KPE 40 mg/kg group showed a significant reduction in final body weight compared with the diabetic control group, although it remained significantly different from the metformin-treated positive control. In contrast, no significant difference in weight gain was observed between the KPE 40 mg/kg group and the metformin-treated group, indicating a comparable effect on weight gain during the experimental period.

Regarding organ weight, skeletal muscle mass was markedly reduced in the diabetes control group compared with the NC group, reflecting diabetes-related muscle loss. Treatment with KPE significantly preserved skeletal muscle weight in a dose-dependent manner, with the 40 mg/kg group showing the greatest improvement (*p* < 0.05). These findings indicate that KPE not only mitigates excessive weight gain but also contributes to the preservation of skeletal muscle mass in diabetic mice.

### 2.5. KPE Improves Glucose Tolerance and Reduces Insulin Resistance in HFD-Fed db/db Mice

**Figure 4:**
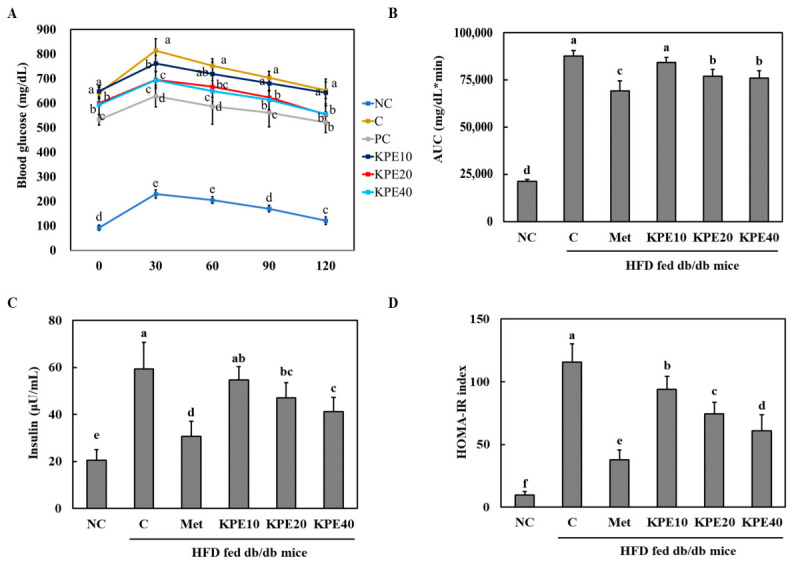
*Kaempferia parviflora* extract (KPE) improves glucose tolerance and insulin resistance in high-fat diet-fed db/db mice. (**A**) Oral glucose tolerance test (OGTT), (**B**) area under the curve (AUC), (**C**) Insulin, and (**D**) HOMA-IR. NC, normal control; C, diabetes-induced control (HFD + db/db mice); Met, HFD + db/db mice + metformin 100 mg/kg body weight [b.w.]; KPE 10, HFD + db/db mice + KPE 10 mg/kg b.w.; KPE 20, HFD + db/db mice + KPE 20 mg/kg b.w.; KPE 40, HFD + db/db mice + KPE 40 mg/kg b.w. Values are expressed as mean ± standard deviation (n = 8). Different superscript letters indicate differences at *p* < 0.05.

Oral glucose tolerance was significantly impaired in the C group compared with the NC, as indicated by elevated blood glucose levels throughout the 120 min OGTT period (Figure 4A). Treatment with KPE improved glucose clearance in a dose-dependent manner. In particular, the KPE 40 mg/kg group showed markedly lower glucose levels at 30, 60, and 120 min compared with the C group, demonstrating enhanced glycemic control.

Consistent with these findings, the area under the curve (AUC) was significantly increased in the C group relative to NC, confirming severe glucose intolerance (Figure 4B). KPE administration reduced AUC values, with the KPE 20 and KPE 40 groups exhibiting significant improvements, approaching the effect observed in the metformin-treated positive control.

Insulin resistance assessed by HOMA-IR was substantially elevated in the C group, whereas all KPE-treated groups showed reduced HOMA-IR indices (Figure 4C). The strongest improvement was observed in the KPE 40 group, which significantly decreased insulin resistance compared with the C group. These results collectively indicate that KPE ameliorates impaired glucose tolerance and improves insulin sensitivity in HFD-fed db/db mice.

### 2.6. KPE Improves Dyslipidemia and Modulates Biochemical Parameters in HFD-Fed db/db Mice

Serum lipid profiles and biochemical parameters were examined to evaluate the metabolic effects of KPE in vivo (Table 2). The C group showed significantly elevated levels of total cholesterol (TC), triglycerides (TG), and LDL-C, along with reduced HDL-C compared with the NC group, indicating pronounced dyslipidemia under diabetic conditions.

KPE supplementation improved lipid metabolism in a dose-dependent manner. Both the KPE 20 and KPE 40 groups exhibited significantly lower TC, TG, and LDL-C levels compared with the C group, with improvements comparable to or exceeding those observed in the Met group. HDL-C levels were also elevated following KPE treatment, with the greatest increase occurring in the KPE 40 group.

Biochemical parameters associated with hepatic function and metabolic regulation were likewise improved by KPE. Elevated AST and ALT levels in the C group were significantly reduced in KPE-treated mice, indicating attenuated hepatic injury. In addition, biomarkers related to oxidative stress and inflammation were ameliorated in the KPE 20 and KPE 40 groups, suggesting broader systemic protective effects.

Collectively, these results indicate that KPE effectively mitigates dyslipidemia and improves metabolic and biochemical abnormalities in HFD-fed db/db mice.

### 2.7. KPE Increases Skeletal Muscle Fiber Size in HFD-Fed db/db Mice

**Figure 5:**
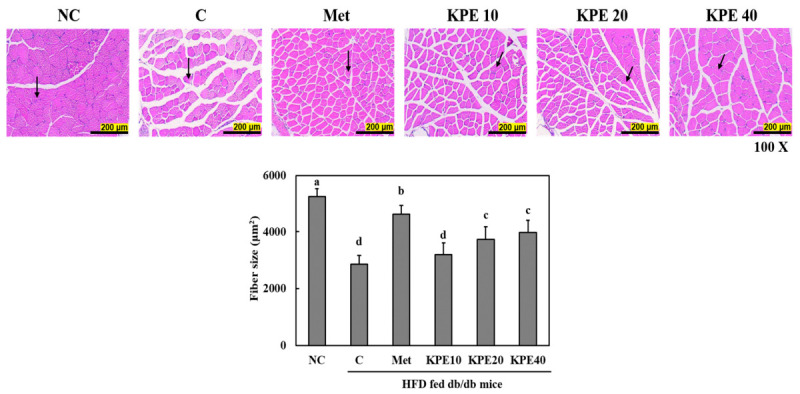
*Kaempferia parviflora* extract (KPE) increases muscle fiber size in the gastrocnemius muscle of high-fat diet-fed db/db mice. NC, normal control; C, diabetes-induced control (HFD + db/db mice); Met, HFD + db/db mice + metformin 100 mg/kg body weight [b.w.]; KPE 10, HFD + db/db mice + KPE 10 mg/kg b.w.; KPE 20, HFD + db/db mice + KPE 20 mg/kg b.w.; KPE 40, HFD + db/db mice + KPE 40 mg/kg b.w. Values are expressed as mean ± standard deviation (n = 8). Different superscript letters indicate differences at *p* < 0.05. Black arrows indicate the measured muscle fibers.

Histological examination of skeletal muscle revealed marked reductions in muscle fiber size in the C group compared with the NC group, indicating substantial muscle atrophy under diabetic and high-fat diet conditions (Figure 5). Treatment with KPE attenuated this atrophy in a dose-dependent manner.

Quantitative analysis showed that muscle fiber cross-sectional area was significantly reduced in the C group, whereas the KPE-treated groups exhibited progressively larger fiber sizes. Among the treated groups, KPE 40 produced the greatest increase in muscle fiber area, approaching the values observed in the Met group. KPE 20 also resulted in a significant improvement compared with the C group, while KPE 10 showed a modest but measurable increase.

These findings demonstrate that KPE effectively mitigates muscle wasting and promotes the preservation of skeletal muscle structure in HFD-fed db/db mice.

### 2.8. KPE Activates Insulin-Signaling Pathways and Enhances Glucose Metabolism in the Skeletal Muscle of HFD-Fed db/db Mice

**Figure 6:**
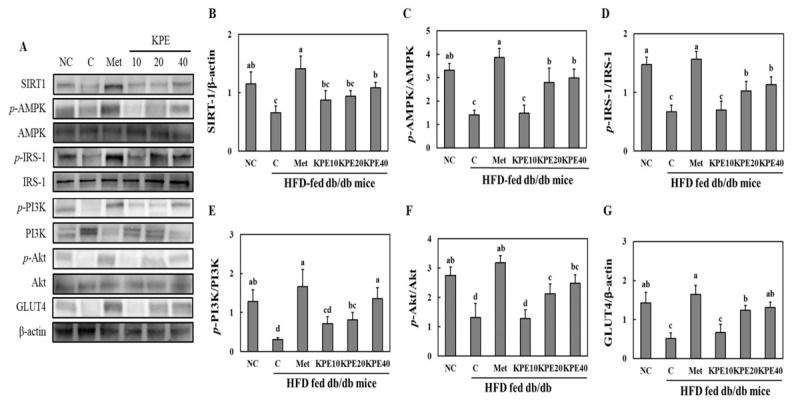
*Kaempferia Parviflora* extract (KPE) enhances glucose uptake and upregulates GLUT4 expression in the gastrocnemius muscle of high-fat diet-fed db/db mice. (**A**) Protein expression, (**B**) SIRT1 (**C**) *p*-AMPK/AMPK (**D**) *p*-IRS-1/IRS-1, (**E**) *p*-PI3K/PI3K, (**F**) *p*-Akt/Akt, and (**G**) GLUT4. NC, normal control; C, diabetes-induced control (HFD + db/db mice); Met, HFD + db/db mice + metformin 100 mg/kg body weight [b.w.]; KPE 10, HFD + db/db mice + KPE 10 mg/kg b.w.; KPE 20, HFD + db/db mice + KPE 20 mg/kg b.w.; KPE 40, HFD + db/db mice + KPE 40 mg/kg b.w. Values are expressed as mean ± standard deviation (n = 8). Different superscript letters indicate differences at *p* < 0.05.

To investigate the molecular mechanisms underlying the glucose-lowering effects of KPE, key proteins involved in insulin signaling and glucose metabolism were examined in skeletal muscle (Figure 6). The C group exhibited markedly reduced expression of SIRT1, *p*-AMPK/AMPK, *p*-IRS-1/IRS-1, *p*-PI3K/PI3K, *p*-Akt/Akt, and GLUT4 compared with the NC group, indicating substantial impairment of insulin signaling and glucose uptake under diabetic conditions.

KPE supplementation restored these signaling pathways in a dose-dependent manner. SIRT1 levels were significantly increased in the KPE 20 and KPE 40 groups compared with the C group, approaching values observed in the Met group. Similarly, phosphorylation of AMPK and IRS-1 was markedly enhanced by KPE treatment, with the KPE 40 group showing the most pronounced improvement.

Activation of downstream metabolic regulators was also recovered by KPE. Phosphorylation of PI3K and Akt—critical mediators of insulin-stimulated glucose uptake—was substantially elevated in the KPE 20 and KPE 40 groups, indicating enhanced insulin responsiveness. GLUT4 expression, which was strongly suppressed in the C group, was significantly upregulated by KPE in a dose-dependent manner, suggesting improved GLUT4-mediated glucose transport capacity in skeletal muscle.

Overall, these data demonstrate that KPE enhances skeletal muscle glucose metabolism by activating the SIRT1/AMPK and IRS-1/PI3K/Akt signaling pathways and restoring GLUT4 expression in HFD-fed db/db mice.

## 3. Discussion

This study demonstrates that KPE exerts significant metabolic benefits in both in vitro and in vivo models of insulin resistance. Across differentiated C2C12 myotubes and HFD-fed db/db mice, KPE improved glucose uptake, enhanced insulin signaling, modulated key metabolic pathways, and ameliorated physiological dysfunction associated with obesity and diabetes. These findings collectively support the potential of KPE as a functional nutraceutical that targets multiple mechanisms underlying metabolic disease.

A central finding of this research is the inhibitory effect of KPE on α-amylase and α-glucosidase, enzymes responsible for starch digestion. Inhibition of these enzymes delays carbohydrate absorption and mitigates postprandial hyperglycemia [20]. The activity observed at higher concentrations of KPE approached that of acarbose, a clinically established α-glucosidase inhibitor [21]. Botanical extracts rich in polyphenolic compounds frequently exhibit similar inhibitory patterns [22], and our results add KPE to the growing list of natural enzyme modulators with antidiabetic potential. Supporting this, previous study reported that KPE fractions containing 5,7-dimethoxyflavone and 4′,5,7-trimethoxyflavone exhibited α-glucosidase inhibitory activity, attributing the effect to the abundance of polymethoxyflavones (PMFs) and plant sterol within the extract [23]. Although digestive enzyme inhibition may not fully explain the in vivo glycemic improvements, it represents an important complementary mechanism contributing to glucose homeostasis.

In lipid-induced insulin-resistant C2C12 myotubes, KPE significantly restored glucose uptake and reversed the impaired GLUT4 expression induced by palmitic acid exposure. These improvements were associated with the activation of key insulin signaling molecules, including IRS-1, PI3K, and Akt [24]. Metformin, as a positive control in Figure 3, has been previously shown to enhance the phosphorylation of IRS-1, PI3K, and Akt, thereby stimulating GLUT4-mediated glucose transport in skeletal muscle [25,26]. The insulin signaling cascade is essential for GLUT4 expression and glucose uptake in skeletal muscle, and its disruption is a hallmark of insulin resistance [24,27]. KPE-mediated restoration of this pathway suggests a direct enhancement of insulin responsiveness in muscle cells, which likely translates to improved systemic glycemic control observed in vivo.

AMPK and SIRT1, two master regulators of cellular energy and metabolic homeostasis, were also significantly upregulated by KPE. AMPK activation promotes glucose uptake, fatty acid oxidation, and mitochondrial function, whereas SIRT1 regulates mitochondrial biogenesis, redox balance, and metabolic adaptation [28,29,30,31]. These findings align with our previous investigation, in which the same standardized KPE activated the AMPK/CPT/UCP1-driven thermogenic pathway in HFD-fed mice and 3T3-L1 adipocytes [32]. The present study extends the relevance of this energy-sensing axis from adipose tissue to skeletal muscle under diabetic conditions. Consistent with earlier work by Kim et al., KPE-mediated SIRT1/AMPK activation likely converges on PGC-1α. The SIRT1-stimulating activity of KPE has been directly demonstrated in vitro, where its major PMF constituents—3,5,7,3′4′-pentamethoxyflavone and 5,7,4′-trimethoxyflavone—showed potent SIRT1 enzyme-stimulating activity, supporting the mechanistic relevance of SIRT1 activation observed in the present study [33]. A master regulator of mitochondrial biogenesis, providing a mechanistic explanation of the improvements in glucose disposal, lipid handling, and muscle preservation observed in this study [19]. Furthermore, our results demonstrated that KPE significantly upregulated the expression of serum GLP-1. As a major therapeutic target for diabetes, GLP-1 is traditionally recognized as an incretin hormone that enhances insulin secretion; however, recent studies emphasize its extra-pancreatic roles, including the direct stimulation of glucose uptake in skeletal muscle. The elevation of GLP-1 by KPE suggests a synergistic enhancement of the SIRT1/AMPK axis, potentially positioning KPE as a natural modulator of GLP-1-mediated metabolic pathways [34,35].

In the in vivo study, db/db mice displayed severe hyperglycemia, poor glucose tolerance, and elevated HOMA-IR, consistent with their genetic predisposition toward obesity and diabetes [36]. KPE supplementation significantly ameliorated these metabolic impairments. Notably, the highest dose (40 mg/kg) produced effects approaching the efficacy of metformin, particularly in lowering glucose levels during the OGTT and reducing AUC. All KPE groups exhibited improved HOMA-IR, with the most pronounced improvements in the 20 and 40 mg/kg groups, indicating enhanced whole-body insulin sensitivity and glucose homeostasis. These results are in line with prior animal studies that have established KP’s antidiabetic potential. Akase et al. demonstrated that KP suppressed insulin resistance, glucose intolerance and hyperinsulinemia in obese T2DM mice [37]. Similarly, a previous study reported that KPE improved glucose tolerance and reduced insulin resistance in HFD-fed diabetic NSY mice, attributing these effects to PPARγ ligand-binding activation and suppression of ectopic lipid accumulation in the liver and skeletal muscle [38]. Consistent with these findings, another study also showed that KPE ameliorated insulin resistance, hyperglycemia, and fatty liver in spontaneously obese TSOD mice [39]. Interestingly, KPE supplementation caused a parallel downward shift in the GTT curve rather than significantly altering the slope of glucose clearance. This kinetic pattern can be attributed to two complementary mechanisms. First, the molecular improvements observed in skeletal muscle—specifically the activation of the IRS-1/PI3K/Akt pathway and increased GLUT4 expression—facilitated enhanced basal glucose uptake. While a steeper decline in the GTT curve is often considered a hallmark of acute muscle-specific clearance, the parallel shift observed here is still highly consistent with improved muscle function within the db/db model. Second, KPE may have suppressed hepatic gluconeogenesis, thereby reducing hepatic glucose output. The concurrent improvement in fasting glucose and increased HDL-cholesterol levels observed in KPE-treated groups suggest enhanced liver function. This is consistent with the previous report that KPE reduced not only insulin resistance but also fatty liver, and that structurally related flavonoids such as *kaempferol* suppress hepatic gluconeogenesis by inhibiting pyruvate carboxylase and glucose-6-phosphatase activity, thereby ameliorating fasting hyperglycemia [39,40]. The extreme hyperglycemia and severe β-cell dysfunction characteristic of these mice limit their acute insulin secretory response, thereby constraining changes in postprandial curve kinetics regardless of peripheral sensitivity. Consequently, the overall reduction in blood glucose reflects the successful restoration of muscle glucose transport and hepatic glucose homeostasis, supported by the significant activation of AMPK/SIRT1-mediated pathways and GLUT4 expression.

KPE also exerted favorable effects on lipid metabolism. Dyslipidemia—manifested as elevated total cholesterol, triglycerides, and LDL-C with reduced HDL-C—is strongly linked to insulin resistance and cardiovascular risk [41,42]. KPE supplementation corrected these abnormalities in a dose-dependent manner. AMPK activation likely contributes to these improvements by stimulating lipid oxidation and suppressing lipogenesis [43]. These lipid-lowering effects are consistent with our previous findings demonstrating that KPE inhibited lipogenesis-related enzyme (ACC, FAS) while promoting lipolysis-related enzyme (ATGL/HSL) in HFD-induced obese mice. These findings are also supported by reports by Lee et al. showing similar correction of dyslipidemia in HFD-fed C57BL/6 mice [32,44]. Extending these findings to humans, previous study demonstrated in a 12-week randomized, double-blind, placebo-controlled trial that daily intake of PMF purified from KP significantly reduced visceral fat area in Japanese overweight adults compared to placebo, without adverse events [45]. Together, these studies indicate that KPE’s lipid-regulatory effects are reproducible across multiple obesity and diabetic models. KPE supplementation produced a marked elevation in HDL-C concentrations, which may represent an additional mechanism contributing to the improvement in fasting glucose. Drew et al. demonstrated in a placebo-controlled crossover study in patients with T2DM that short-term HDL-C elevation significantly reduced plasma glucose via AMPK activation in skeletal muscle through an ABCA1/CaMKK-dependent pathway, as well as through enhanced pancreatic β-cell insulin secretion [46]. These mechanisms are consistent with the AMPK activation and improved insulin sensitivity observed in KPE-treated groups in the present study, suggesting that HDL-C elevation may serve as a complementary pathway through which KPE exerts its antidiabetic effects. Additionally, elevated liver enzymes (AST and ALT) in diabetic mice were attenuated by KPE, suggesting a hepatoprotective effect and improved metabolic integrity at the organ level. These hepatoprotective findings are consistent with previous report demonstrating that KPE significantly reduced liver weight, hepatic TG and TC accumulation in TSOD diabetic mice, indicating an improvement in fatty liver conditions [39]. The attenuation of elevated serum AST and ALT levels in KPE-treated diabetic mice is likely attributable to both direct and indirect hepatoprotective mechanisms of its PMFs constituents. Directly, previous studies have shown that PMFs-rich extracts modulate the Nrf/AMPK signaling pathway to suppress oxidative stress and inflammatory cytokines, thereby preventing hepatocellular membrane damage and reducing hepatocellular leakage into circulation [47]. Indirectly, KPE-derived PMFs improve insulin sensitivity via PPARγ regulation and attenuate advanced glycation end products (AGE) formation under hyperglycemic conditions, collectively alleviating the upstream metabolic burden on the liver [48]. These findings suggest that the observed improvement in liver enzyme profiles reflects a multifaceted hepatoprotective action of KPE at both the cellular and systemic metabolic levels.

Morphological analysis of skeletal muscle revealed pronounced muscle fiber atrophy in db/db mice, a common consequence of chronic metabolic dysfunction [10,49]. Muscle mass is critical for systemic glucose disposal, as skeletal muscle accounts for the majority of insulin-stimulated glucose uptake [50,51]. KPE supplementation restored muscle fiber size, particularly at higher doses, indicating improved muscle preservation. These findings are consistent with a previous study demonstrating that KPE markedly increased muscle fiber cross-sectional area (CSA), muscle volume, and mass of the gastrocnemius, tibialis anterior, and soleus muscle in ob/ob mice, accompanied by activation of the PI3K/Akt/mTOR pathway and suppression of the muscle atrophy markers atrogin-1 and MuRF-1 [52]. These structural benefits likely arise from enhanced insulin signaling, improved mitochondrial function through upregulation of key mitochondrial biogenesis regulators, including PGC-1α, nuclear respiratory factor-1 (NRF-1), and mitochondrial transcription factor A (Tfam), as previously demonstrated in L6 myotubes treated with KPE [53]. Notably, the increase in muscle fiber CSA observed in the KPE-treated groups closely correlated with the changes in muscle weights recorded in Table 1. In particular, the significant attenuation of weight loss in the gastrocnemius muscle by KPE aligns with the hypertrophic changes seen in our histological assessment. While the total mass of the quadriceps and TA muscles showed a more gradual recovery, the consistent improvement in individual fiber CSA demonstrates that KPE effectively prevents the atrophic remodeling of myocytes. These findings suggest that KPE does not merely increase bulk muscle mass but specifically enhances the structural quality and integrity of skeletal muscle by reversing the lipid-induced catabolic state associated with diabetes.

Molecular analyses further corroborated these improvements. KPE enhanced phosphorylation of IRS-1, PI3K, and Akt, reinforcing the restoration of insulin signaling in skeletal muscle. Increased expression of GLUT4 supports improved glucose uptake capacity [54]. Together, these findings provide a coherent molecular basis for the observed reductions in fasting glucose, AUC, and HOMA-IR. The combination of enzyme inhibition, improved insulin signaling, and AMPK/SIRT1 activation highlights KPE as a multifaceted regulator of metabolic homeostasis.

The bioactivity of KPE is largely attributed to its PMFs, such as 5,7-dimethoxyflavone and 5,7,4′-trimethoxyflavone [32,55]. A comprehensive review cataloged approximately 141 chemical constituents from *Kaempferia* species, highlighting that KPE is uniquely characterized by a rich diversity of PMFs, including 5,7-dimetholxyfalvone, 3,5,7-trimethoxyflavone, and 3,5,7,4′-tetramethoxyflavone, which collectively underpin its broad-spectrum pharmacological activities [56]. These compounds have been previously reported to activate AMPK, reduce inflammation, and enhance mitochondrial function [57,58,59,60]. Although HPLC profiling was not performed in the present study, the KPE used here was prepared from the same extract and using the same protocol as our previous anti-obesity investigation, in which 5,7-dimethoxyflavone was identified as a representative marker compound [32]. Therefore, it is reasonable to consider that the metabolic improvements observed in this study could be mediated in part by these previously characterized PMFs. Our data support these prior findings and further extend them by demonstrating that KPE also preserves muscle morphology and improves systemic metabolic parameters in a diabetic mouse model.

The comparative analysis between Metformin and KPE in this study provides meaningful clinical insights. Metformin, the gold-standard first-line treatment for type 2 diabetes, demonstrated superior potency in lowering fasting blood glucose at a relatively lower dose (100 mg/kg) and is highly effective for rapid glycemic control [61]. In contrast, KPE at 40 mg/kg b.w. showed a multifaceted advantage by simultaneously modulating a broader range of signaling molecules, including SIRT1 and GLP-1, alongside AMPK. While Metformin is a potent therapeutic agent, KPE—as a natural botanical extract—offers a holistic approach with potentially fewer gastrointestinal side effects, making it an attractive candidate for long-term preventive intervention [6]. However, KPE requires a higher dosage to match the glucose-lowering efficacy of Metformin.

While our findings are robust, several limitations must be considered. First, although db/db mice represent a well-established model of type 2 diabetes, human physiologic responses may differ. Clinical studies evaluating KPE supplementation in humans, including dose–response assessments, pharmacokinetics, and long-term safety, are warranted to translate these findings into practical dietary or therapeutic recommendations. Additionally, while AMPK and SIRT1 activation were observed, the specific molecular targets of KPE and its individual flavonoids remain unclear. Further mechanistic studies are needed to determine whether KPE interacts directly with upstream kinases, modulates NAD^+^ availability, or influences oxidative stress pathways. The synergistic potential of KPE with existing antidiabetic drugs also merits exploration. Combination therapies that target distinct metabolic pathways may yield enhanced outcomes compared with monotherapies. Furthermore, the antioxidant and metabolic regulatory properties of KPE suggest possible benefits in preventing diabetes-related complications, such as neuropathy, sarcopenia, and hepatic steatosis.

In conclusion, this study provides compelling evidence that KPE ameliorates metabolic dysfunction by targeting multiple complementary pathways. KPE modulates multiple metabolic pathways, improves skeletal muscle insulin signaling, enhances GLUT4 expression, activates AMPK and SIRT1, and preserves muscle morphology. These coordinated effects contribute to improved glucose tolerance, reduced insulin resistance, and corrected dyslipidemia in HFD-fed db/db mice. Taken together, these results highlight the potential of KPE as a functional food ingredient or nutraceutical for managing insulin resistance and type 2 diabetes. Continued research into its mechanisms, optimal dosing, and clinical applicability will further clarify its role in metabolic disease prevention and intervention.

## 4. Materials and Methods

### 4.1. Preparation of KPE

KPE used in this study was identical to the standardized extract described in our previous publication [32]. Briefly, dried rhizomes of KP were extracted with hydrous ethanol, followed by filtration, concentration, and drying to obtain the powdered extract. The extract had been previously standardized to contain more than 4% 5,7-dimethoxyflavone using high-performance liquid chromatography (HPLC). Since the same batch and preparation protocol were employed in the present study, detailed phytochemical profiling was not repeated here.

### 4.2. Measurement of α-Glucosidase and α-Amylase Inhibitory Activities

α-glucosidase and α-amylase inhibitory activities of KPE were assessed using commercial colorimetric assay kits (Biovision, Milpitas, CA, USA) [61]. KPE was tested at concentrations ranging from 20 to 100 μg/mL, and acarbose was included as a positive control. For the α-glucosidase assay, 10 μL of enzyme solution (1.0 U/mL) was added to each well containing KPE, followed by assay buffer to a final volume of 50 μL. The reaction was initiated by adding 50 μL of substrate mix, and absorbance was measured at 410 nm using a microplate reader (Bio-Rad, Hercules, CA, USA).

For the α-amylase assay, 5 μL of α-amylase (3 U/mL) was added to KPE-containing wells, and the volume was adjusted to 50 μL with distilled water. A reaction mixture containing substrate solution (100 μL total volume) was then added, and absorbance was recorded at 405 nm. All assays were performed according to the manufacturer’s instructions, and inhibitory activity was calculated relative to the enzyme-only control.

### 4.3. C2C12 Cell Culture, Differentiation and Treatments

C2C12 myoblasts (ATCC, Rockville, MD, USA) were cultured in high-glucose DMEM (Hyclone, Logan, UT, USA) supplemented with 10% fetal bovine serum (Gibco, Carlsbad, CA, USA), 1% penicillin–streptomycin, 1% L-glutamine, and 1% sodium pyruvate under standard conditions (37 °C, 5% CO_2_). For differentiation, cells were seeded at a density of 1 × 10^6^ cells per well in 6-well plates and allowed to reach full confluence. The medium was then replaced with DMEM containing 2% horse serum (Gibco) and 10 nM insulin (Sigma-Aldrich, St. Louis, MO, USA) and maintained for 5 days to induce myotube formation. To evaluate the preventive effect of KPE, the treatment was initiated during the differentiation process. Specifically, from day 3 to day 5 of differentiation, cells were co-treated with 0.5 mM palmitic acid (PA) and various concentrations of KPE or metformin (1 mM) every 24 h. Insulin resistance was induced by treating cells with PA in differentiation medium [62].

Cells were then assigned to the following experimental groups: normal control (NC), untreated myotubes; PA-treated control (C), myotubes exposed to PA in 2% horse serum medium; Met, PA-treated myotubes co-treated with metformin (1 mM); and KPE-treated groups, PA-treated myotubes supplemented with *Kaempferia parviflora* extract at 20, 50, or 100 μg/mL (KPE 20, KPE 50, KPE 100, respectively). After treatment for the indicated time period, cells were harvested for glucose uptake assays and Western blot analysis.

### 4.4. Glucose Uptake Measurement in Differentiated C2C12 Myotubes

Glucose uptake was assessed in differentiated C2C12 myotubes using a colorimetric 2-deoxy-D-glucose (2-DG) uptake assay kit (BioVision, Milpitas, CA, USA), following the manufacturer’s instructions. After differentiation and sample treatments as described above, cells were washed twice with warm Krebs–Ringer–phosphate–HEPES (KRPH) buffer (pH 7.4) containing 2% bovine serum albumin (BSA), followed by a 40 min incubation at 37 °C. To evaluate insulin-stimulated glucose uptake, the cells were then stimulated with 1 μM insulin for 20 min. Following insulin stimulation, cells were exposed to 10 mM 2-DG for 20 min at 37 °C. The reaction was terminated by rapidly washing cells with ice-cold PBS, and intracellular 2-DG uptake was quantified through an enzymatic cycling reaction that generates NADPH. Absorbance was measured at 412 nm using a microplate reader (iMark™, Bio-Rad, Hercules, CA, USA). Glucose uptake was normalized to cellular protein content, and all experiments were performed in triplicate [63].

Glucose uptake was assessed in differentiated C2C12 myotubes using a colorimetric 2-deoxy-D-glucose (2-DG) uptake assay kit (BioVision, Milpitas, CA, USA), following the manufacturer’s instructions. After differentiation and sample treatments as described above, cells were washed twice with warm Krebs–Ringer–phosphate–HEPES (KRPH) buffer (pH 7.4) containing 2% bovine serum albumin (BSA), followed by a 40 min incubation at 37 °C. To evaluate insulin-stimulated glucose uptake, the cells were then stimulated with 1 μM insulin for 20 min. Following insulin stimulation, cells were exposed to 10 mM 2-DG for 20 min at 37 °C. The reaction was terminated by rapidly washing cells with ice-cold PBS, and intracellular 2-DG uptake was quantified through an enzymatic cycling reaction that generates NADPH. Absorbance was measured at 412 nm using a microplate reader (iMark™, Bio-Rad, Hercules, CA, USA). Glucose uptake was normalized to cellular protein content, and all experiments were performed in triplicate [63].

### 4.5. Experimental Animals and Treatments

All animal procedures were approved by the Institutional Animal Care and Use Committee of Kyung Hee University (KHGASP-24-576). Male C57BL/6J mice (6 weeks old, 22.6 ± 0.57 g) and male C57BLKS/J-db/db mice (6 weeks old, 40.81 ± 2.27 g) were obtained from Saeronbio, Inc. (Uiwang, Republic of Korea). A total of 48 mice were used in this study (n = 8 per group). Animals were housed in a specific pathogen-free barrier facility at 23 ± 3 °C and 55% relative humidity, under a 12 h light/dark cycle, with ad libitum access to food and water. After a 1-week acclimation period on a standard chow diet, the mice were randomly assigned to six experimental groups. To evaluate the preventive potential of KPE against the rapid escalation of diabetic phenotypes, treatment was initiated at 7 weeks of age. The normal control group consisted of C57BL/6J mice maintained on the AIN-93G diet (containing 17.2% kcal from fat), whereas all db/db mice were fed a 60% high-fat AIN-93G-based diet (containing 60% kcal from fat) (both diets from Research Diet, Inc., New Brunswick, NJ, USA) to induce chronic metabolic stress, mirroring the PA-induced lipotoxicity used in our in vitro C2C12 model. Among the db/db groups, one served as the diabetic control, while another received metformin (100 mg/kg) via oral gavage as a positive control to benchmark the activation of the AMPK pathway in this preventive setting. The remaining db/db mice were administered KPE at three different doses (10, 20, or 40 mg/kg) daily by oral gavage to evaluate dose-dependent effects.

Body weight, food intake, and fasting blood glucose levels were recorded weekly to monitor the progression of metabolic dysfunction. At the end of the experimental period, mice were fasted for 4 h and euthanized. This short-term fasting duration was selected to represent a physiological basal state rather than a state of total starvation, as previously demonstrated, allowing assessment of basal insulin signaling in peripheral tissues while avoiding the metabolic stress associated with prolonged fasting [64]. Skeletal muscle tissues were collected, snap-frozen in liquid nitrogen, and stored at −80 °C until further analyses [61].

### 4.6. Oral Glucose Tolerance Tests (OGTTs)

In the final week of the experimental period, all mice were fasted for 4 h prior to testing. A glucose solution (2 g/kg body weight) was administered orally, and blood samples were collected from the tail vein at 0, 30, 60, 90, and 120 min following glucose loading. Blood glucose concentrations were measured using the G. Doctor Blood Glucose Monitoring System (Allmedicus, Anyang, Republic of Korea). The area under the curve (AUC) was calculated to assess glucose tolerance [61].

### 4.7. Biochemical Analysis of Blood Samples

At the end of the experimental period, whole blood was collected following euthanasia. Serum was obtained by centrifugation at 3000 rpm for 20 min at 4 °C and used for biochemical analysis. Serum glucose levels were measured using a Glucose Assay Kit (BioVision, Milpitas, CA, USA). Serum insulin concentrations were quantified using a Mouse Insulin ELISA Kit (Crystal Chem Inc., Elk Grove Village, IL, USA). Glycated hemoglobin (HbA1c) levels were determined using a Mouse Hemoglobin A1c Kit (Crystal Chem Inc., USA). Triglycerides (TG), total cholesterol (TC), high-density lipoprotein cholesterol (HDL-C), and low-density lipoprotein cholesterol (LDL-C) were measured using colorimetric assay kits TG/TC/HDL-C/LDL-C Kits, (Abcam, Cambridge, UK) according to the manufacturer’s protocols. Serum aspartate aminotransferase (AST) and alanine aminotransferase (ALT) activities were measured using commercially available colorimetric kits (AST/ALT Activity Kits, Abcam, Cambridge, UK). Additionally, glucagon-like peptide-1 (GLP-1) levels were assessed using a Mouse GLP-1 ELISA Kit (Elabscience, Houston, TX, USA), and dipeptidyl peptidase-IV (DPP-IV) activity was quantified using a DPP-IV Activity Assay Kit (Abcam, Cambridge, UK). All biochemical measurements were performed in duplicate to ensure analytical accuracy.

### 4.8. Western Blotting

Total protein was extracted from differentiated C2C12 myotubes and mouse skeletal muscle tissues using CelLytic™ MT Cell Lysis Reagent (Sigma-Aldrich, St. Louis, MO, USA) supplemented with Halt™ Protease and Phosphatase Inhibitor Cocktail (Thermo Fisher Scientific, Rockford, IL, USA). For tissue samples, approximately 25 mg of skeletal muscle was homogenized in 0.5 mL of lysis buffer. Homogenates were centrifuged at 14,000× *g* at 4 °C for 15–20 min, and the supernatants were collected for analysis. Protein concentrations were determined using the Bradford assay.

Equal amounts of protein (20–40 μg) were separated on 10% Mini-PROTEAN^®^ TGX™ precast gels (Bio-Rad, Hercules, CA, USA) and transferred onto PVDF membranes using the Trans-Blot^®^ Turbo™ Transfer System (Bio-Rad). Membranes were blocked with 5% skim milk prepared in Tris-buffered saline containing 0.1% Tween-20 (TBST) for 1 h at room temperature, followed by overnight incubation at 4 °C with primary antibodies against SIRT1 (#9475), AMPK (#2532), phospho-AMPK (#2513S), IRS-1 (#2382), phospho-IRS-1 (#2381), PI3K (#4292S), phospho-PI3K (#4228), Akt (#9272), phospho-Akt (#9271), GLUT4 (#2213) were purchased from Cell Signaling Technology (Danvers, MA, USA) and β-actin (A300-491A) was purchased from Bethyl Laboratories, which were used at 1:500–1:1000 dilutions.

After washing, membranes were incubated with horseradish peroxidase-conjugated anti-rabbit or anti-mouse secondary antibodies (1:2000) for 1 h at room temperature. Protein bands were visualized using EzWestLumi Plus chemiluminescent substrate (ATTO, Tokyo, Japan) and detected with the Ez-Capture II imaging system (ATTO). Band intensities were quantified using CS Analyzer 3.0 software (ATTO) [61].

### 4.9. Histological Study

Skeletal muscle tissues collected from mice at sacrifice were fixed in 10% (*v*/*v*) neutral-buffered formalin for 48 h and subsequently transferred to 70% ethanol for tissue processing. Fixed tissues were embedded in paraffin, and 5 μm thick sections were prepared using a rotary microtome for histological evaluation. For H&E staining, tissue sections were deparaffinized in xylene and rehydrated through a graded ethanol series. Slides were stained with hematoxylin for 10 min, rinsed in running tap water, and differentiated as needed before counterstaining with eosin for 3 min. After staining, slides were dehydrated through graded ethanol and cleared in xylene. All stained tissues were mounted using ProLong™ Gold antifade reagent (Thermo Fisher Scientific, Waltham, MA, USA). Morphological changes and muscle fiber size were examined under an Olympus BX40F4 microscope (Southern Microscope Inc., Haw River, NC, USA), and representative images were captured for analysis [64]. To determine muscle fiber size, the CSA was measured using Image J software (version 1.54k). To minimize confounding errors arising from different sectioning angles, only fibers cut in a strictly transverse orientation were selected for measurement, while obliquely sectioned or elongated fibers were excluded.

### 4.10. Statistical Analysis

Data are presented as the mean ± standard deviation (SD). Statistical comparisons among groups were performed using one-way analysis of variance (ANOVA), followed by Duncan’s multiple range test for post hoc evaluation. All analyses were conducted using IBM SPSS Statistics version 29 (IBM Corp., Armonk, NY, USA). A *p* value of less than 0.05 was considered indicative of statistical significance.

## Figures and Tables

**Table 1:** Impact of KPE administration on body weight, weight gain, and organ weights.

	Groups		High-Fat Diet-Fed db/db Mice				
Variables		NC	C	Met	KPE 10	KPE 20	KPE 40
Initial body weight (g)		22.58 ± 0.57 ^b^	40.81 ± 2.27 ^a^	38.62 ± 4.30 ^a^	39.78 ± 4.53 ^a^	40.56 ± 2.78 ^a^	39.98 ± 3.09 ^a^
Final body weight (g)		27.97 ± 0.93 ^c^	67.16 ± 1.67 ^a^	63.58 ± 1.39 ^b^	65.52 ± 1.59 ^a^	63.34 ± 1.60 ^b^	62.09 ± 1.69 ^c^
Weight gain (g) *		5.39 ± 0.87 ^c^	26.35 ± 2.85 ^a^	24.96 ± 4.51 ^ab^	25.74 ± 3.30 ^ab^	22.78 ± 2.07 ^ab^	22.21 ± 2.10 ^b^
Water intake (mL/day)		3.48 ± 0.46 ^c^	12.16 ± 0.71 ^a^	10.03 ± 0.79 ^b^	11.37 ± 0.68 ^a^	10.30 ± 0.80 ^a^	10.44 ± 1.03 ^a^
Organ weight (g)							
Liver		1.15 ± 0.10 ^e^	3.97 ± 0.30 ^a^	3.46 ± 0.29 ^c^	3.87 ± 0.32 ^ab^	3.52 ± 0.48 ^bc^	2.93 ± 0.33 ^d^
Kidney		0.34 ± 0.03 ^c^	0.70 ± 0.09 ^a^	0.60 ± 0.05 ^b^	0.58 ± 0.08 ^b^	0.52 ± 0.08 ^b^	0.56 ± 0.06 ^b^
Spleen		0.09 ± 0.01 ^b^	0.12 ± 0.01 ^a^	0.10 ± 0.01 ^ab^	0.11 ± 0.01 ^ab^	0.10 ± 0.02 ^b^	0.09 ± 0.02 ^b^
Skeletal muscle (g)							
Quadriceps		0.30 ± 0.04 ^a^	0.14 ± 0.02 ^b^	0.17 ± 0.02 ^b^	0.15 ± 0.02 ^b^	0.16 ± 0.02 ^b^	0.16 ± 0.02 ^b^
Tibialis anterior (TA)		0.16 ± 0.02 ^a^	0.07 ± 0.01 ^b^	0.08 ± 0.01 ^b^	0.08 ± 0.02 ^b^	0.08 ± 0.01 ^b^	0.08 ± 0.01 ^b^
Gastrocnemius		0.33 ± 0.02 ^a^	0.15 ± 0.02 ^d^	0.21 ± 0.01 ^b^	0.17 ± 0.01 ^cd^	0.17 ± 0.01 ^c^	0.18 ± 0.02 ^c^

NC, normal control; C, diabetes-induced control (HFD + db/db mice); Met, HFD + db/db mice + metformin 100 mg/kg body weight [b.w.]; KPE 10, HFD + db/db mice + KPE 10 mg/kg b.w.; KPE 20, HFD + db/db mice + KPE 20 mg/kg b.w.; KPE 40, HFD + db/db mice + KPE 40 mg/kg b.w. Values are presented as mean ± standard deviation (n = 8), and different superscript letters indicate significance at *p* < 0.05. * Weight gain (g/6 weeks) = final body weight (g) − initial body weight (g).

**Table 2:** Effect of *Kaempferia parviflora* extract (KPE) on blood lipid profiles and biochemical parameters in high-fat diet-fed db/db mice.

	Groups		High-Fat Diet-Fed db/db Mice				
Variables		NC	C	Met	KPE 10	KPE 20	KPE 40
AST (mU/mL)		41.59 ± 5.32 ^c^	71.54 ± 4.15 ^a^	52.30 ± 7.16 ^b^	49.31 ± 6.14 ^b^	52.81 ± 5.01 ^b^	49.95 ± 6.69 ^b^
ALT (mU/mL)		12.40 ± 1.99 ^d^	56.90 ± 3.04 ^a^	37.70 ± 7.81 ^c^	44.71 ± 3.27 ^b^	48.79 ± 4.61 ^b^	44.88 ± 8.13 ^b^
TG (mM)		1.37 ± 0.39 ^d^	4.84 ± 0.75 ^a^	3.55 ± 0.43 ^c^	4.57 ± 0.56 ^ab^	4.11 ± 0.58 ^bc^	3.88 ± 0.30 ^c^
TC (μg/μL)		1.19 ± 0.24 ^d^	2.35 ± 0.28 ^a^	1.81 ± 0.21 ^bc^	2.10 ± 0.22 ^ab^	2.01 ± 0.26 ^bc^	1.78 ± 0.23 ^c^
LDL-C (μg/μL)		0.34 ± 0.09 ^d^	2.32 ± 0.51 ^a^	1.41 ± 0.28 ^bc^	1.79 ± 0.45 ^b^	1.30 ± 0.35 ^c^	1.14 ± 0.33 ^c^
HDL-C (μg/μL)		0.91 ± 0.15 ^d^	1.45 ± 0.20 ^cd^	2.52 ± 0.44 ^ab^	1.98 ± 0.45 ^bc^	2.97 ± 0.39 ^a^	3.26 ± 0.26 ^a^
LDL/HDL ratio		0.32 ± 0.08 ^d^	1.47 ± 0.19 ^a^	0.58 ± 0.05 ^c^	0.97 ± 0.16 ^b^	0.46 ± 0.16 ^cd^	0.33 ± 0.08 ^d^
Glucose (nmol/μL)		10.93 ± 1.96 ^e^	40.93 ± 3.11 ^a^	27.76 ± 1.05 ^d^	38.74 ± 2.56 ^ab^	35.73 ± 2.56 ^bc^	33.25 ± 3.48 ^c^
HbA1c (%)		4.79 ± 0.45 ^e^	10.72 ± 1.16 ^a^	8.03 ± 0.74 ^d^	10.21 ± 0.93 ^ab^	9.37 ± 0.46 ^bc^	8.98 ± 0.77 ^c^
GLP-1 (pmol/L)		20.54 ± 4.89 ^a^	7.35 ± 1.24 ^c^	12.55 ± 3.53 ^b^	8.15 ± 3.03 ^c^	11.35 ± 2.48 ^bc^	12.95 ± 1.96 ^b^
DPP-IV (ng/L)		0.25 ± 0.03 ^d^	0.59 ± 0.07 ^a^	0.42 ± 0.04 ^bc^	0.48 ± 0.04 ^b^	0.48 ± 0.07 ^b^	0.39 ± 0.07 ^c^

NC, normal control; C, diabetes-induced control (HFD + db/db mice); Met, HFD + db/db mice + metformin 100 mg/kg body weight [b.w.]; KPE 10, HFD + db/db mice + KPE 10 mg/kg b.w.; KPE 20, HFD + db/db mice + KPE 20 mg/kg b.w.; KPE 40, HFD + db/db mice + KPE 40 mg/kg b.w. Values are presented as mean ± standard deviation (n = 8), and different superscript letters indicate significance at *p* < 0.05.

## Data Availability

The original contributions presented in this study are included in the article. Further inquiries can be directed to the corresponding authors.

## Abbreviations

2-DG	2-deoxy-D-glucose
Akt	protein kinase B
ALT	Alanine Aminotransferase
AST	Aspartate Aminotransferase
AUC	The area under the curve
BSA	Bovine Serum Albumin
C	Control group (differentiated C2C12 myotubes with palmitic acid treatment in vitro; high-fat diet-fed db/db mice in vivo)
DMEM	Dulbecco’s Modified Eagle Medium
DPP-4	Dipeptidyl Peptidase-4
GLP-1	Glucagon-Like Peptide-1
HbA1c	Hemoglobin A1c
HDL-C	High-Density Lipoprotein Cholesterol
HFD	high fat diet
HOMA-IR	Homeostasis Model Assessment of Insulin Resistance
HS	Horse serum
IRS-1	insulin receptor substrate-1
KPE	*Kaempferia parviflora* rhizome extract
LDL-C	Low-Density Lipoprotein Cholesterol
Met	Metformin
NC	Normal Control (differentiated C2C12 myotubes without palmitic acid treatment in vitro; AIN-93G-fed C57BL/6J mice in vivo)
OGTTs	Oral glucose tolerance tests
PA	palmitic acid
PMFs	polymethoxyflavones
PI3K	phosphoinositide 3-kinase
SIRT1	Sirtuin 1
T2DM	type 2 diabetes mellitus
